# Eyes-Closed Increases the Usability of Brain-Computer Interfaces Based on Auditory Event-Related Potentials

**DOI:** 10.3389/fnhum.2018.00391

**Published:** 2018-09-28

**Authors:** David Hübner, Albrecht Schall, Natalie Prange, Michael Tangermann

**Affiliations:** ^1^Brain State Decoding Lab, Department of Computer Science, University of Freiburg, Freiburg, Germany; ^2^Cluster of Excellence, BrainLinks-BrainTools, Freiburg, Germany

**Keywords:** eyes-open, eyes-closed, usability, event-related potentials, auditory, brain-computer interface, P300, EEG

## Abstract

Recent research has demonstrated how brain-computer interfaces (BCI) based on auditory stimuli can be used for communication and rehabilitation. In these applications, users are commonly instructed to avoid eye movements while keeping their eyes open. This secondary task can lead to exhaustion and subjects may not succeed in suppressing eye movements. In this work, we investigate the option to use a BCI with eyes-closed. Twelve healthy subjects participated in a single electroencephalography (EEG) session where they were listening to a rapid stream of bisyllabic words while alternatively having their eyes open or closed. In addition, we assessed usability aspects for the two conditions with a questionnaire. Our analysis shows that eyes-closed does not reduce the number of eye artifacts and that event-related potential (ERP) responses and classification accuracies are comparable between both conditions. Importantly, we found that subjects expressed a significant general preference toward the eyes-closed condition and were also less tensed in that condition. Furthermore, switching between eyes-closed and eyes-open and vice versa is possible without a severe drop in classification accuracy. These findings suggest that eyes-closed should be considered as a viable alternative in auditory BCIs that might be especially useful for subjects with limited control over their eye movements.

## 1. Introduction

In the last decade, brain-computer interfaces (BCIs) relying on auditory stimuli experienced a burst of activity (Nijboer et al., 2008; Furdea et al., 2009; Klobassa et al., 2009; Halder et al., 2010, 2016; Schreuder et al., 2010, 2011; Höhne et al., 2011, 2012; Lopez-Gordo et al., 2012; Käthner et al., 2013; Nambu et al., 2013; Pokorny et al., 2013; De Vos et al., 2014; Kindermans et al., 2014; Tangermann et al., 2014, 2018; Simon et al., 2015; Baykara et al., 2016; Real et al., 2016; Xiao et al., 2016; Zhou et al., 2016; Hübner and Tangermann, 2017). In these BCIs, the subject hears different tones or natural sounds (e.g., animal sounds, syllables or words) while the subjects brain signals are recorded, e.g., with electroencephalography (EEG). Using machine learning methods (Blankertz et al., 2001; Wolpaw et al., 2002; Dornhege et al., 2007; Lemm et al., 2011; Wolpaw and Wolpaw, 2012), a computer can process these signals to predict which of the stimuli was attended (a so-called *target*) and which was ignored (a *non-target*).

Different applications are based on this information. The most widespread use-case is to allow paralyzed patients, e.g., those with amyotrophic lateral sclerosis (ALS), a neurodegenerative muscle disease, to communicate on a very basic level (Wolpaw et al., 2002; Sellers and Donchin, 2006; Münßinger et al., 2010; Zickler et al., 2013). In this scenario, each stimulus is associated with a control command, e.g., a patient responds with “yes” by attending a high tone and can say “no” by attending a low tone. Another application field of auditory BCIs is the brain-state assessment of patients with disorders of consciousness (Pokorny et al., 2013; Real et al., 2016; Xiao et al., 2016). These approaches explore the idea that BCIs can detect residual brain activity even for patients where it is unknown whether they are conscious. Recently, we have shown that auditory BCIs can also be used for language training after stroke (Tangermann et al., 2018).

A problem that is typically encountered when recording brain activity by means of EEG is the occurrence of artifacts. Although most subjects have fewer problems to suppress body movements, *eye artifacts* such as blinks or eye movements are hard to eliminate during the measurement and their associated EEG signals are much stronger than the brain signals of interest. This is especially challenging for subjects wearing contact lenses (leading to dry eyes) and for the – often elderly – patients. Blinking rates were shown to be influenced by the workload (Van Orden et al., 2001) which is often quite high in BCI experiments.

In our BCI-based language training (Tangermann et al., 2018), we experienced one case where a chronic stroke patient was unable to voluntarily reduce the number of eye blinks. The subject was persistently blinking about once every second. This led to an extremely deteriorated quality of the EEG recordings. Different methods have been developed to alleviate the effects of eye artifacts using linear regression methods (Parra et al., 2005) or independent component analysis (ICA) decompositions (Fatourechi et al., 2007; Winkler et al., 2011, 2014), but they still lead to a significant data loss and cannot perfectly separate eye artifacts from underlying brain activity.

Additionally, the unnatural instruction to avoid eye blinks for a prolonged period constitutes for an unwanted secondary task that is distracting the subject from the main task and typically involves a substantial level of stress. This can have the undesired consequence that a training based on EEG signals is less efficient due to the split of cognitive resources to the main training task and to the secondary task of avoiding eye blinks. In an extreme scenario, subjects may spend so much attention on suppressing eye artifacts, that they are unable to perform the main task.

The difficulty of avoiding eye movements over a long period leads to the question if the number of eye artifacts could be reduced and the measurement can be made more comfortable for the test subject by having the subject *close their eyes while collecting the data*. This idea is feasible in auditory BCIs since visual input is not needed during a trial. In this study, we will compare two conditions: eyes-closed (**EC**) and eyes-open (**EO**). While many studies have shown that EC leads to an increase in occipital alpha as well as a changed topology and activity in different frequency bands compared to EO (see Barry et al., 2007), the existing literature—to our knowledge—lacks an analysis of the EC condition for event-related potentials (ERPs) in the fast paradigms that are used for BCIs.

These ERPs are voltage deflections that are the results of the brain processing an event (such as hearing a tone or a word). Many BCIs rely on the P300 component which describes a positive voltage deflection that occurs after 250–400 ms in an oddball task where an infrequent target tone (e.g., a high tone) is played among frequent non-target tones (e.g., low tones). The P300 is thought to be produced by a distributed network of brain processes associated with attention and subsequent memory operations (Polich, 2007). The temporal delay between the onsets of two sounds is called stimulus onset asynchrony (SOA) and is known to modulate the P300 amplitude and latency (Höhne and Tangermann, 2012). An older study by Intriligator and Polich found that the “P300 amplitude is relatively unaffected by the factor [whether the eyes are open or closed]” in a two-tone oddball task with an SOA of 1 s (Intriligator and Polich, 1994). A more recent meta-review comes to the same conclusion stating that latency and amplitude of the P300 were not significantly different between EO and EC in the standard oddball task with an SOA of 1 s and tones as stimuli (van Dinteren et al., 2014). Remarkably, several hundred subjects were included in this meta-analysis for each condition (*N*_*EO*_ = 555, *N*_*EC*_ = 998) where the data was collected from several studies (16 studies used EO and 23 studies used EC).

However, results from this meta-analysis are not directly transferable to BCIs as (a) the SOA between two stimuli in the meta-review (1 s) is much longer than in recent BCIs (typically SOAs vary between 250-550ms) and because (b) tone stimuli lead to different ERP responses compared to natural (more complex) sounds (animals sounds or words) that are used in modern BCIs (Höhne et al., 2012; Tangermann et al., 2014, 2018; Simon et al., 2015; Baykara et al., 2016; Halder et al., 2016). In addition, questions regarding (c) the number of eye artifacts and (d) user comfort and usability were not investigated.

Another relevant research question is whether a system trained on data recorded with EO could be applied when the subject has their eyes closed and the other way around. If this is the case, subjects could switch between conditions within one session. This could be expected to improve the overall comfort of the subject during the measurement and decrease the stress level.

In summary, this study should investigate four main hypotheses.

**H1:** EC leads to fewer eye artifacts than EO.
**H2:** The achieved target vs non-target classification accuracies do not differ significantly between EO and EC.
**H3:** The measuring process is overall more comfortable for the subjects for EC than for EO.
**H4:** A system trained on data recorded in one condition can be applied in the other condition without a substantial loss in classification accuracy.

## 2. Materials and methods

In a within-subject design, we compared the EEG signals and usability aspects for the conditions EC and EO in an auditory BCI paradigm using words as stimuli and a fast SOA of 250 ms.

### 2.1. Participants

Twelve healthy volunteers [11 subjects between 22 and 29 (mean = 25.2 years, *SD* = 2.04 years), and one subject (S7) aged 76, 5 female in total] were recruited for the experiment. All 12 subjects reported having normal hearing. Following the Declaration of Helsinki, approval for this study was obtained by the ethics committee of the University Medical Center Freiburg and all participants gave written informed consent prior to participation. A session took about 3.5 h (including the EEG set-up and washing the hair).

### 2.2. Experimental structure and stimuli

**Figure 1:**
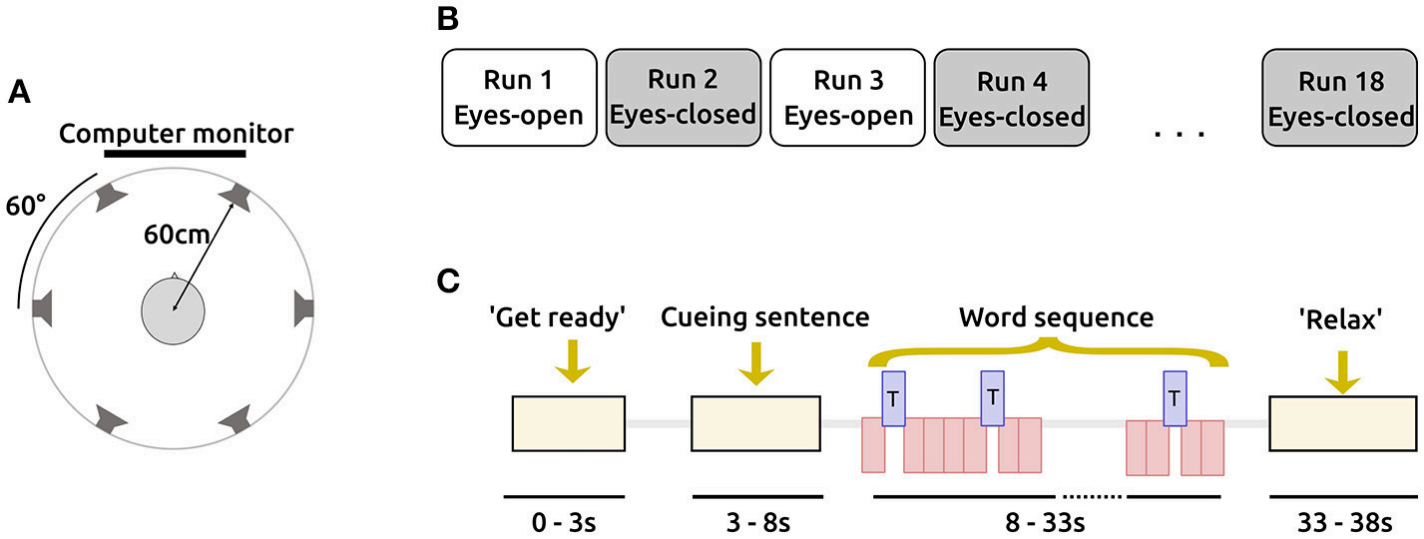
Structure and design of the study. **(A)** AMUSE setup in a top view. Six loudspeakers are spatially centered around the subject's head. Figure adapted from Schreuder et al. (2010). **(B)** A session consisted of 18 runs alternating between eyes-open and eyes-closed. Each run consists of 6 trials. **(C)** A trial comprises 4 distinct stages. The timings (in seconds) indicate the beginnings of each stage. During the word sequence, targets (T; blue) and non-targets (red) are interleaved and played with a fast SOA of 250 ms.

Subjects were asked to be seated comfortably on a chair, facing a computer monitor. Six loudspeakers were centered in 60° steps, at ear height around the subjects head, with a radius of approximately 60 cm (see Figure 1A). The auditory stimuli were presented from the six loudspeakers according to the AMUSE (Auditory MUlticlass Spatial ERP) paradigm (Schreuder et al., 2010).

A session consisted of a total number of 18 runs, each contained six trials. Runs where subjects had their eyes closed were followed by runs where subjects had their eyes open and vice versa (see Figure 1B) to alleviate effects of non-stationaritiy in the EEG signals. The current condition was indicated to the user on the screen. To prevent systematic errors, the condition used in the first run alternated between participants.

In each trial, one out of six bisyllabic words (length = 300 ms) were cued by a sentence as target stimuli before presenting a sequence of word stimuli (SOA = 250 ms), see also Figure 1C. In a familiarization phase before the EEG recording, these sentence-word mappings were practiced with the subjects. During the sequence, each speaker played a different distinct word 15 times, resulting in a class-wise ratio of 1:5, with 15 target and 75 non-target stimuli per trial. Per condition (EO/EC), 9 runs were recorded. As each of them contains 6 trials, our experiment resulted in 54 trials per condition. Multiplying these 54 trials per condition with the number of targets per trials (15) and the number of non-targets per trial (75) results in a total of 810 targets and 4050 non-targets per subject and condition (EO/EC), respectively. In a run, each of the six stimuli was chosen exactly once as a target, while the other stimuli served as non-targets. We pseudo-randomized the ordering in which the stimuli were presented and in which the targets were selected. The mapping from stimulus to loudspeaker was also performed pseudo-randomized.

### 2.3. Data acquisition and processing

The study consisted of the EEG recordings during the AMUSE paradigm and the subjective ratings mainly after the EEG measurements.

#### 2.3.1. Subjective ratings

For both conditions (EO / EC), we assessed several subjective ratings after the session in a questionnaire. Subjects were asked to rate their ergonomic experience during the EEG recordings for eight items regarding motivation, concentration, fatigue, eye movement suppression, eye blink suppression, stimulus discrimination, exhaustion, and difficulty of the task on a 5-point Likert scale. We also asked the subject which condition they preferred overall (EO / EC / undecided). We further used the Self-Assessment Manikin (SAM) (Bradley and Lang, 1994), which is a non-verbal pictorial assessment technique, to assess valence from 1 (negative) to 9 (positive), and arousal from 1 (calm) to 9 (excited). In addition, we asked the subjects to indicate their general fatigue before and after the EEG measurement on a 5-point Likert scale.

#### 2.3.2. EEG data acquisition

EEG activity was recorded and amplified by a multichannel EEG amplifier (BrainAmp DC, Brain Products) and with 63 passive Ag/AgCl electrodes (EasyCap). The channels were placed according to the 10-20-system, referenced against the nose and grounded at channel AFz. Electrode impedances were kept below 15 kΩ. Eye signals were recorded by Electrooculography (EOG) with an electrode below the right eye of a subject (the channel associated with this electrode is hereafter called EOGvu). The signal was sampled at a rate of 1 kHz.

In addition to the EEG and EOG channels, pulse (on an index finger) and respiration (diaphragmatic breathing) were recorded, but not further analyzed.

#### 2.3.3. EEG data preprocessing

The offline analysis of the EEG data was performed using the BBCI toolbox (Blankertz et al., 2010). The data was bandpass filtered in [0.5 8] Hz using a Chebyshev Type II filter and downsampled to 100 Hz. EEG signals were then epoched between –200 and 1,200 ms relative to the stimulus onset. A baseline correction was then performed based on data within the interval [-200, 50] ms.

We marked those epochs where the difference of the highest and lowest value in one epoch exceeded 60 μV in one of the frontal channels (Fp1, Fp2, F7, F8, F9, F10) to capture eye- or other muscular artifacts. We call this step *Minmax*_60. The percentage of epochs that gets flagged by this procedure (and by additional steps that will be described below) is reported in the result section. In total, we applied three different preprocessing pipelines in addition to the steps mentioned before:

**P1:** Only the above steps were applied. We call this condition *Minmax*_60.
**P2:** Before applying *Minmax*_60, we estimated the horizontal eye movements based on the bipolar channel EOGh, which is defined as the difference between the channels F9 and F10, and the vertical eye movement based on the bipolar channel EOGv, which is the difference between the channels Fp2 and EOGvu. By assuming a stationary eye movement pattern, the regression approach from Parra et al. (2005) was then used to project out the eye artifacts. In addition, channels showing very little variance (less than 0.5 μV in more than 10% of the trials) or too much variance (more than 3 times the difference between the 90^th^ percentile and the 10^th^ percentile of the variance of all channels) were rejected. Moreover, trials with very high variance (similar to before, trials with variance exceeding 3 times the difference between the 90^th^ percentile and the 10^th^ percentile of the variance of all trials) were also flagged. We call the variance-related treatment of artifacts as variance criterion (short *Var*).
**P3:** We applied the Multiple Artifact Rejection Algorithm, short MARA (Winkler et al., 2011, 2014), a supervised machine learning algorithm to reject eye components that is based on independent component analysis (ICA). Its core is 1290 expert-labeled ICA components that are used to automatically classify ICA components as being artifacts or not. ICA components that have been classified as artifacts are then projected out. This approach has been shown to reliably detect eye artifacts even on unseen data (Winkler et al., 2014).

#### 2.3.4. Classification

Per EEG channel, the amplitudes were averaged in eight intervals ([100, 190], [191, 300], [301, 450], [451, 560], [561, 700], [701, 850], [851, 1000] and [1001, 1200] ms). These intervals have shown good classification results in our previous studies that used the same auditory BCI protocol. They had been handcrafted to capture the time intervals with the highest discriminatory power for typical subjects. We fixed them in the study design before recording the data to avoid a potential overfitting to the obtained classification accuracies. This strategy resembles the situation in an online experiment, where no classification parameters can be changed *post-hoc*. Of course, and most importantly, the selected interval boundaries were the same for the two conditions (EO/EC) to guarantee a fair comparison. For visualization (Figure 5), we manually picked those intervals that show the most discriminatory time intervals after computing the grand averages.

This led to a 504-dimensional feature vector (63 channels with 8 intervals each) per epoch. The classification between target and non-target stimuli was performed using a Linear Discriminant Analysis (LDA) classifier with shrinkage-regularized covariance matrix (Blankertz et al., 2011). The LDA classifier can be understood as a hyperplane separating the multidimensional feature space into binary classes. The shrinkage regularization allows the LDA classifier to gain good classification results even in the case of high feature dimensionality and a low number of data points. An estimation of the class-wise means and (regularized) covariance matrices was computed using the samples within subjects. If not specified further, we applied a five-fold chronological cross-validation for estimating the classification accuracies. Accuracies are reported as area under the curve (AUC) of the receiver-operator curve. The AUC values can range between 0 and 1, with a theoretical chance level of 0.5. An AUC value of 1 indicates a perfect separation between targets and non-targets. The AUC can be understood as the probability that a target receives a higher score by the classifier compared to a non-target.

## 3. Results

### 3.1. Hypothesis 1 (eye artifacts)

**Figure 2:**
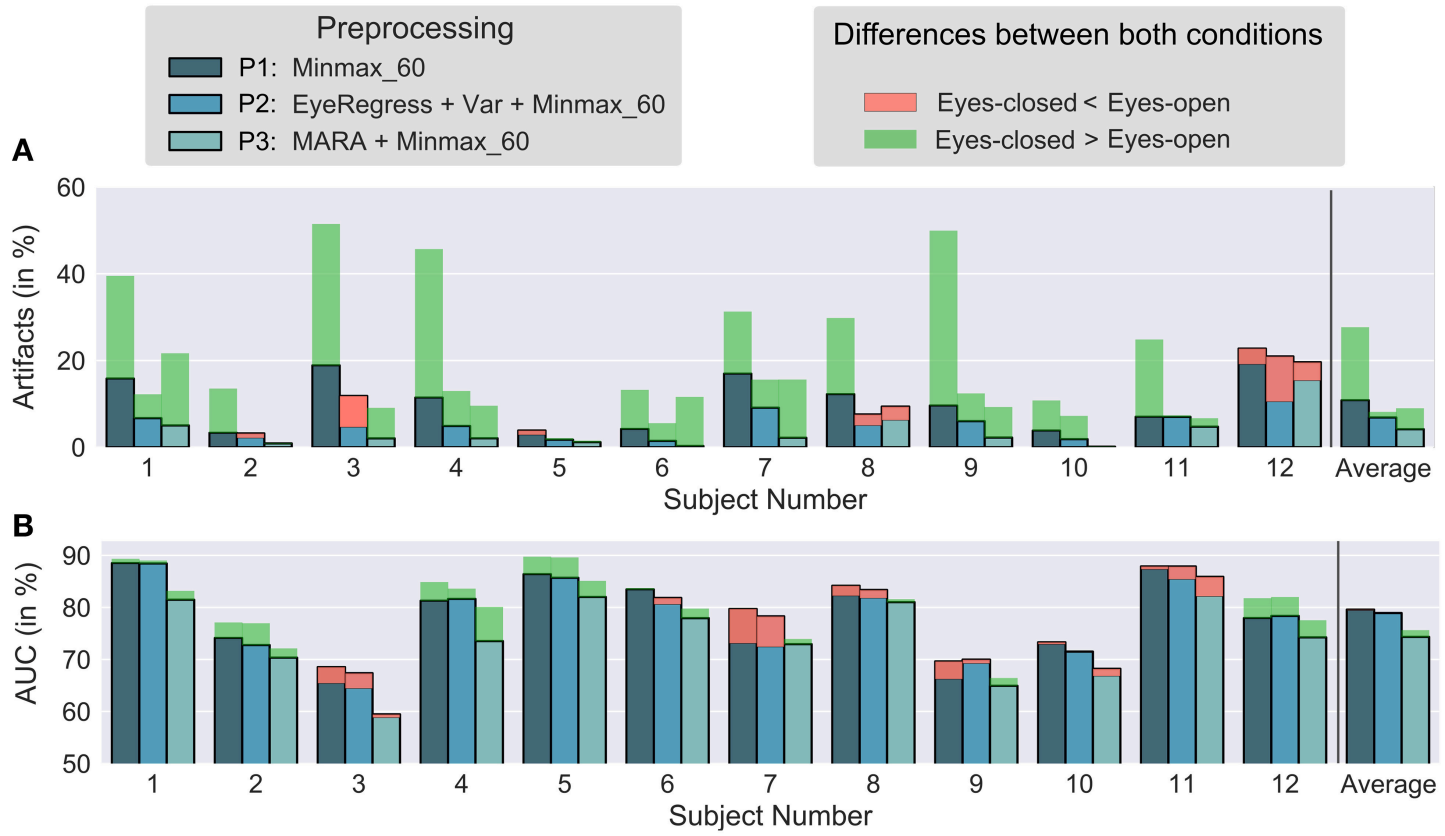
Number of artifacts and classification accuracies for different preprocessing methods. **(A)** The percentage of artifacts obtained by *Minmax*_60 (and the variance criterion in case of P2) for all subjects. **(B)** Cross-validated classification accuracy for all subjects. The solid blue-ish bars depict the smaller value for the two conditions (EO/EC). The red or green bars indicate the value of that condition which led to a higher outcome.

In order to test whether the EC condition leads to fewer artifacts than the EO condition, we applied three different preprocessing pipelines (P1-P3) to the data as explained in the method section. The results are shown in Figure 2A. By visual inspection, one can observe that the number of artifacts is higher for the EC condition. A Wilcoxon signed rank test over the percentage of artifact trials for each participant for EO and EC shows that the number of artifacts is significantly higher for EC when only *Minmax*_60 is applied (**P1**: *W* = 3, *p* = 0.0024), but not for the other two preprocessing condition (**P2**: *W* = 29, *p* = 0.5; **P3**: *W* = 9, *p* = 0.037) when applying the Bonferroni-Holm correction (uncorrected p-values are reported). Hence, the hypothesis that there are less artifact trials in the EC condition could not be confirmed. Given the very consistent results, it is unlikely that more subjects will deliver different results. Instead, the data suggests the opposite, namely, that more eye artifacts exist with EC compared to EO.

### 3.2. Hypothesis 2 (accuracy)

We examined whether the accuracies differ between EO and EC. Depending on the preprocessing and condition, the grand average performance was around 75–80% (see Figure 2B). The Wilcoxon signed-rank test was used to test the null hypothesis that the accuracies are the same for both conditions. We found that for all three preprocessing pipelines, there was no significant different between the two conditions (**P1**: *W* = 38, *p* = 0.9, **P2**: *W* = 40, *p* = 0.9, **P3**: *W* = 17, *p* = 0.1). It may be the case that a clear trend evolves in the case of measuring a larger number of subjects. However, the small difference between the two groups (the absolute difference between the average performances is less than 1.5 % classification accuracy for all three preprocessing pipelines in our data) and the non-significant result from the meta-review concerning the oddball ERP responses for several hundreds of subjects, convinces us that the effect of the condition on classification accuracy is rather limited.

### 3.3. Hypothesis 3 (usability)

In order to determine whether the measuring process is more comfortable for subjects in the EC condition than in the EO condition, we statistically evaluated a subset of five questions that the participants have answered in the questionnaire.

1. How much did you struggle with fatigue in the different conditions?
2. How easy was it to avoid eye movements in the different conditions?
3-4. How was your mood during the different conditions in terms of valence (negative vs. positive) and arousal (calm vs. tensed)?
5. Overall, which condition did you prefer?

**Figure 3:**
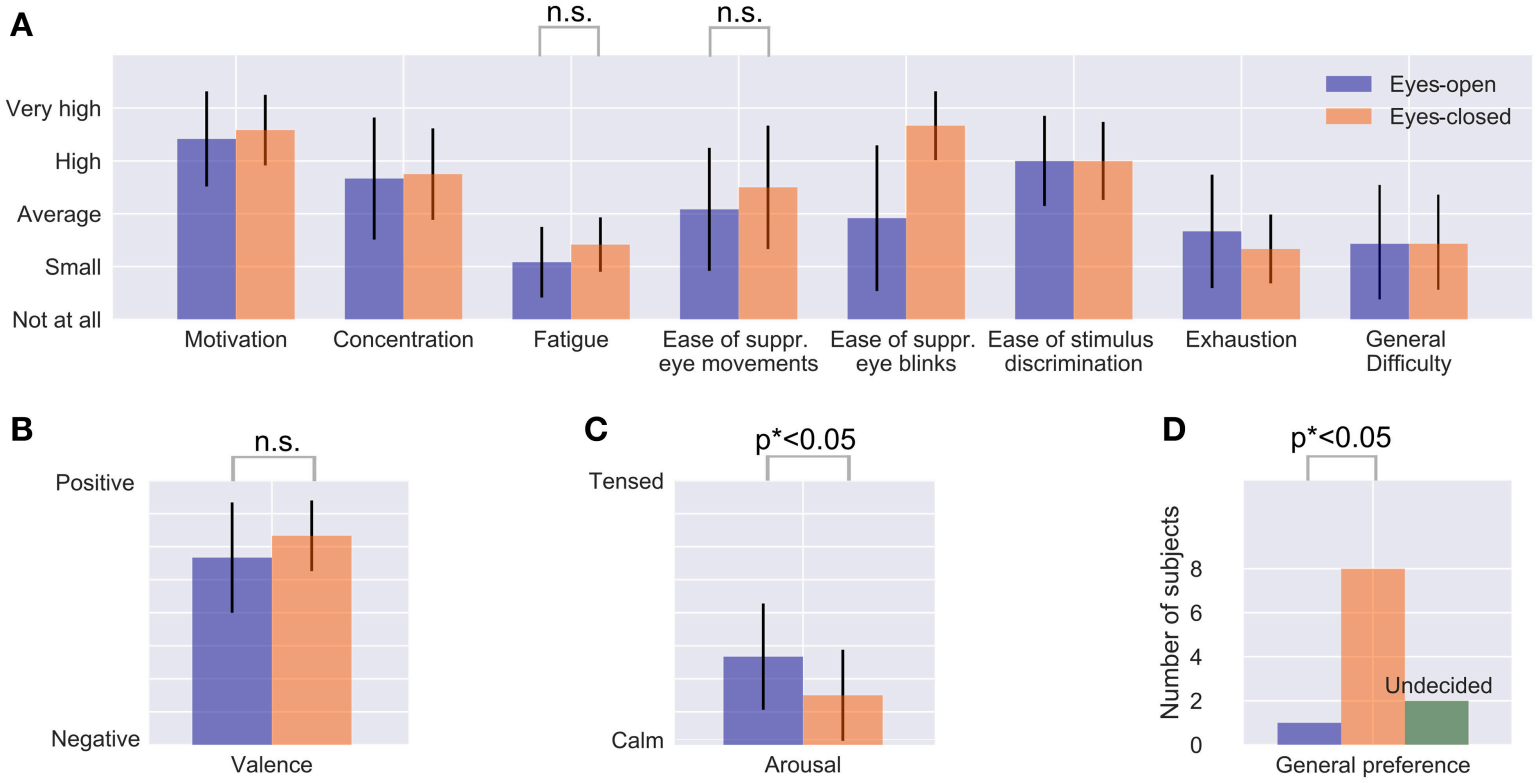
Questionnaire results regarding usability. The mean values and standard deviation of the 12 subjects are shown for each category **(A–D)**. p^*^ indicates Bonferroni-Holm corrected *p*-values, n.s. means “not significant”.

We limited the statistical evaluation to these five questions to reduce the number of multiple comparisons, but report the results for all categories of the questionnaire (see Figure 3). For the five statistical tests, we corrected the resulting p-values with the Bonferroni-Holm correction. A paired *t*-test was applied as it was shown to have the same statistical power as a signed Wilcoxon signed-rank test in case of a 5-point Likert scale (see De Winter and Dodou, 2010).

We found no significant differences for fatigue [*t*_(11)_ = 1.77, *p* = 0.10] and the ease of suppressing eye movements [*t*_(11)_ = 1.16, *p* = 0.27], see Figure 3A. For valence, results suggest that EC was perceived as more positive [*t*_(11)_ = 2.35, *p* = 0.039 (uncorrected)], but this was not significant after Bonferroni-Holm correction (Figure 3B). Significant effects were found for arousal [*t*_(11)_ = 3.92, *p* = 0.002 (uncorrected)] showing that participants were calmer with EC and also for the general preference (Figure 3C). Nine out of twelve subjects preferred EC, only one subject preferred EO and two subjects were undecided. A one-sided binomial test yields *p* = 0.006 (uncorrected), hence we can reject the null hypothesis that both conditions have the same comfort level (Figure 3D). Please find the complete results of the questionnaire in the Supplementary Table S1.

### 3.4. Hypothesis 4 (transferability)

**Figure 4:**
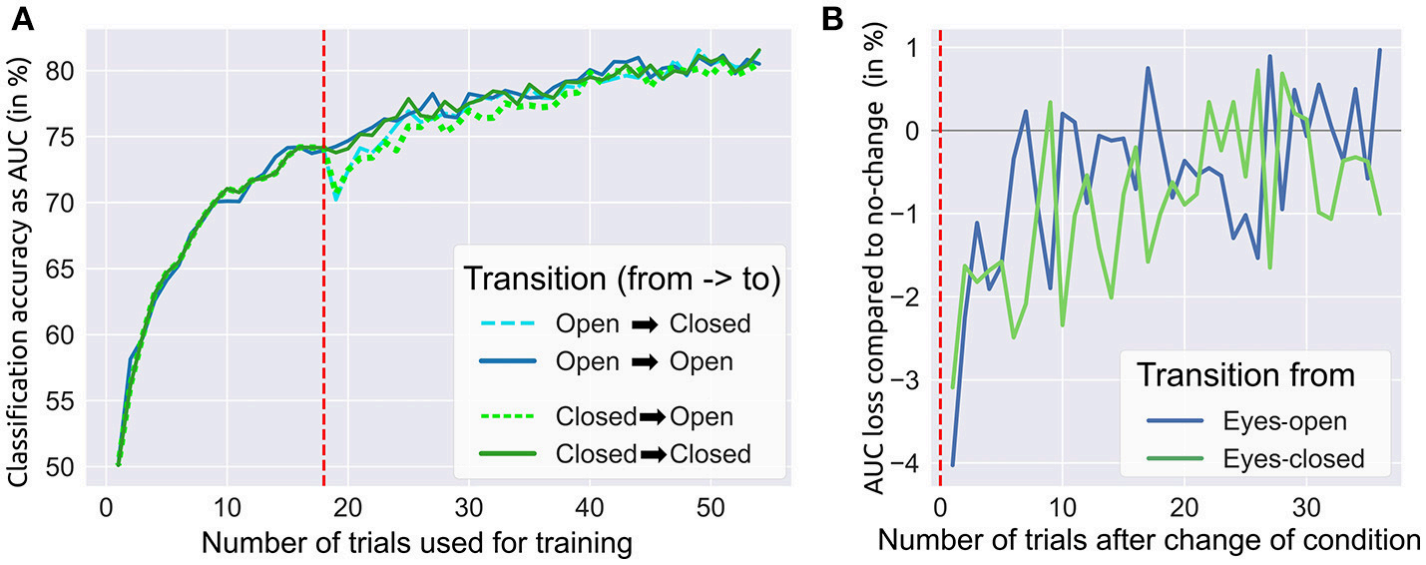
Influence of changing from eyes-closed to eyes-open and vice versa. **(A)** A switch of conditions was simulated after 18 trials (dashed red line) yielding a small reduction in target vs. non-target classification accuracy (measured by AUC). All classifiers were continuously retrained after each trial (see text). **(B)** This subplot shows the loss in accuracy when changing from one condition to the other.

We investigated whether a system trained on data recorded with EO could be applied in runs with EC and vice versa. Therefore, we ran a *post-hoc* offline simulation consisting of two parts. The first part describes the influence of the training set size only, while no transfer learning between conditions was applied. For each subject, we utilized data of the first 18 trials of a condition (EO / EC) to draw an increasing number of randomly chosen trials. Then each of these sets was used to train a shrinkage-regularized LDA classifier. The performance of each classifier was then tested on another randomly selected (but unseen) trial from the same condition and subject. This procedure was repeated many times and with different seeds for the random selection of training and testing data. The average over these repetitions delivered a reliable performance estimate for growing sizes of training data sets. The grand average results are shown in Figure 4A (left to the red dashed line). Both conditions performed very similarly during this part.

In the second part we investigated the effects of transfer learning, i.e., the switching between conditions after 18 trials and continued application on the remaining 36 trials (remember that we had 54 trials per condition in total). Four different transitions were simulated offline: two transitions with a change of conditions (EO → EC, EC → EO) and—to allow for comparisons—another two without a change of conditions (EO → EO, EC → EC). In each of the four scenarios, we took the LDA classifier that was trained on the first 18 EO or EC trials (depending on the condition before the transition). Afterward, we tested the classifier on a randomly drawn trial of the condition after the transition. This trial was then added to the training data and the LDA classifier was retrained on the slightly enlarged training data. As a result of changed conditions, the target vs. non-target accuracy initially dropped around 3–4% (from ~74 % to ~71 %), while no drop was observed when conditions were maintained (see Figure 4A, right to the red dashed line). Collecting and including more data from the condition after the transition, the performance differences between change and no change rapidly decreased until they were not distinguishable anymore after 30 new trials (see Figure 4B). In both phases, we applied the aforementioned randomization procedure with 20 different seeds to obtain reliable results.

### 3.5. ERP analysis

**Figure 5:**
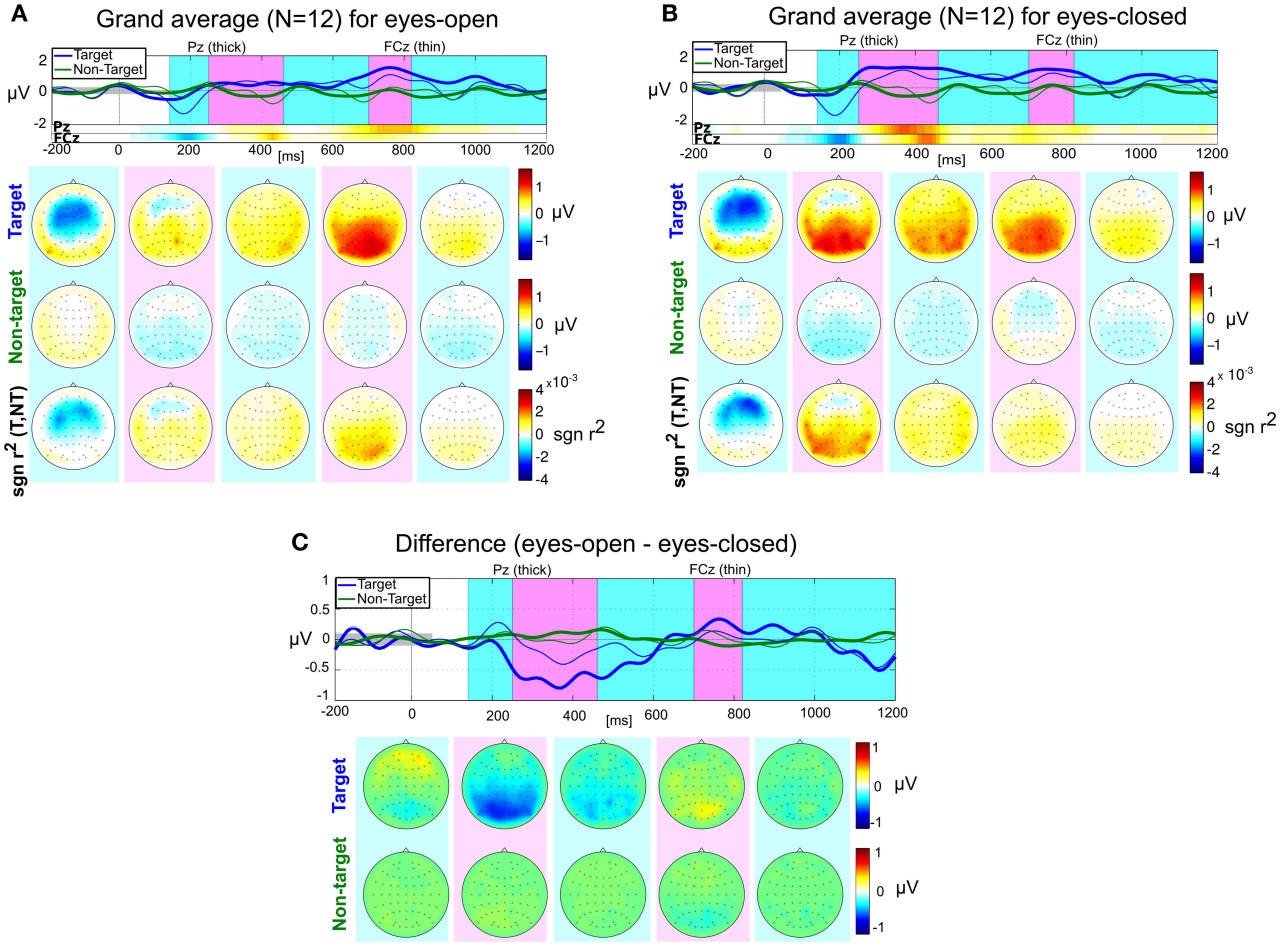
Grand average ERP responses for eyes-open **(A)**, eyes-closed **(B)**, and their differences **(C)**. Top rows: Average responses evoked by target (blue) and non-target (green) stimuli in the central channel Cz (thick) and the fronto-central channel FCz (thin). The signed *r*^2^ values for these two channels are provided by two horizontal color bars. Their scale is identical to the scale of the plots in the bottom row of scalp plots. Target/non-target rows: scalp plots visualizing the spatial distribution of mean target and non-target responses within five selected time intervals: [140, 250], [251, 460], [461, 700], [701, 820], and [821, 1200] ms relative to stimulus onset. Bottom row: scalp plots with signed *r*^2^ values indicate spatial areas with high class-discriminative information.

In addition to the four main hypotheses, we also investigated the shapes, amplitudes and latencies of the ERP responses for both conditions. Figure 5 shows the grand average ERP responses after processing the data with pipeline P2 (although noisy channels were not removed when computing the grand average). The most relevant features (in a linear discriminatory sense) can be inferred from the signed *r*^2^ plots in the bottom row of Figures 5A,B. Two main components are visible for EO and EC: An early negativity with a peak location around FCz and a peak latency of around 200 ms (“N200”) and a later positivity (“P300”) in the parietal area. To quantitatively describe these components, we computed the peak amplitudes and latencies for each subject. The results are presented in Table 1.

**Table 1:** Overview of peak latencies (in ms) and amplitudes (in **μ**V) for the 12 subjects.

	**Eyes-open**				**Eyes-closed**			
	**N200 (FCz)**		**P300 (Pz)**		**N200 (FCz)**		**P300 (Pz)**	
	**Lat**.	**Ampl**.	**Lat**.	**Ampl**.	**Lat**.	**Ampl**.	**Lat**.	**Ampl**.
S1	197	–2.42	792	2.41	206	–1.70	310	2.32
S2	230	–0.65	808	1.53	203	–0.87	428	3.73
S3	150	–0.23	740	0.80	150	–0.50	805	0.81
S4	195	–0.82	796	1.99	197	–0.41	346	2.93
S5	237	–1.85	390	1.81	244	–1.91	437	1.96
S6	169	–0.41	704	1.19	179	–1.04	561	0.89
S7	239	–2.36	615	2.09	250	–2.08	543	2.01
S8	220	–1.27	840	1.86	212	–1.60	512	1.64
S9	195	–2.02	755	1.94	221	–1.46	687	1.91
S10	213	–1.28	359	1.84	223	–1.37	339	1.25
S11	190	–3.53	742	1.59	190	–2.92	570	1.81
S12	179	–1.48	743	1.47	195	–3.23	742	2.11
Mean	201	–1.53	690	1.71	206	–1.59	523	1.95
SD	27.6	0.96	158	0.43	27.4	0.87	161	0.82

*N200 peaks were computed using channel FCz in the interval [140, 280] ms. P300 peaks were calculated from channel Pz in the interval [300, 900] ms. A bootstrapping approach was used to improve the reliability of the peak estimates in which peaks were estimated 10 times on subsets containing randomly-drawn 80% of the data and then averaged across subsets*.

The most striking difference between EO and EC is that the late parietal positivity (P300) appears to be earlier in the EC condition compared to the EO condition, see Figure 5C. A two-sided paired *t*-test for the four quantities (N200 amplitude and latency and P300 amplitude and latency) showed no significant differences between the experimental conditions after Bonferroni-Holm correction, although the P300 latency differs strongly [uncorrected *T*-test, *t*_(11)_ = 2.96, *p* = 0.013] and is likely to become significant with more data points.

## 4. Discussion and conclusion

The goal of this study was to compare the EC and EO condition in a fast auditory BCI paradigm. In brief, our results show that EC leads to comparable signals (with slightly more eye artifacts) while clearly being preferred by the users. Although we have investigated a limited number of subjects only, we observed significant effects which indicate a strong influence of the condition on usability. In the introduction, we mentioned a stroke patient that could not avoid very frequent eye blinks. We instructed this patient to proceed with EC. Afterward, he could successfully control an auditory BCI although he reported to sometimes ‘'drift away,” i.e., to lose focus.

These important findings can have a direct impact on the usability of auditory BCIs. It suggests that subjects should either start with EC right from the beginning or, even better, subjects should simply have the choice to use their preferred condition (EC/EO). This strategy could mitigate major difficulties that are faced when working with subjects that have limited control over their eye movements. In addition, we could show that a transition from one condition to another leads only to a small loss in classification accuracy that quickly diminishes when the classifier is retrained on new data. Especially during longer sessions, we think that this small sacrifice of classification accuracy justifies the improved user comfort.

To understand why condition EC led to an increased number of eye artifacts, we have conducted an additional analysis where we computed the number of artifacts for the two bipolar channels EOGh and EOGv (see preprocessing pipeline P2). These channels should mainly capture horizontal and vertical eye movements, respectively. The analysis shows that eye artifacts in the EC condition originate from vertical as well as from horizontal eye movements with a similar proportion. We believe that the increased number of eye artifacts in the EC condition comes from the absence of a fixation cross. With that, it is rather difficult to not move the eyes and subjects involuntarily produce small saccades. Interestingly, this point has not been reported by the subjects in the questionnaire. Although not significant, they reported that they perceived it as easier to suppress eye movements in the EC condition.

Although not significant, we observed that the P300 peak latency is much larger for the EO condition compared to the EC condition. To explain this observation, we hypothesize that the EO condition has a higher task demand due to the need to simultaneously process visual and auditory input whereas no visual input needs to be processed in the EC condition. This may lead to higher overall workload in the EO condition and thus, explain increased P300 latencies.

We designed the protocol is such a way that EC and EO runs are alternating. The idea behind this design was to reduce the effect of any non-stationarities that occur over the course of a longer session due to human factors (user learning, changed user strategies, fatigue), medication or external factors (drying gel, changed cap position) changing the ERP responses (Shenoy et al., 2006). On the one side, we believe that this design actually led to an underestimation of the severity of eye movements in the EO condition due to the frequent runs where subjects had their eyes closed. One subject remarked that “it would have been difficult to leave the eyes open without the runs where I had my eyes closed.” On the other side, fatigue might become a more severe problem when longer sessions with EC are conducted. We think that the EC strategy should be further tested in real application scenarios to identify possible shortcomings.

A possible limitation with our questionnaire regarding the subjective ratings is that the answers for each item were ordered from unfavorable to favorable, e.g., for the question “How motivating were the different conditions for you?” the possible answers were sorted from “not at all motivating” to “very motivating.” This same ordering for all questions might increase the effect of participants trying to answer consistently. Ordering the possible answers for each item randomly might help to avoid this issue.

Taken together, this is the first study that systematically compares the eyes-closed and eyes-open condition for an auditory BCI. We found that the eyes-closed condition should be considered as a viable alternative to increase the user comfort. In addition, we encourage other scientists and BCI practitioners to test the eyes-closed condition for subjects that fail to control a BCI due to frequent eye movements.

## Data availability statement

The results from the questionnaire can be found in the supplemental data. The raw EEG data sets for this study can be found in the Zenodo Database (doi: 10.5281/zenodo.1298606).

## Author contributions

DH and MT designed the study. MT provided resources to conduct the study. DH, AS, and NP collected and analyzed data. DH wrote the first draft of the paper and AS, NP, and MT revised the manuscript.

### Conflict of interest statement

The authors declare that the research was conducted in the absence of any commercial or financial relationships that could be construed as a potential conflict of interest.
